# Assessment of the Number of Valid Observations and Diurnal Changes in Chl-a for GOCI: Highlights for Geostationary Ocean Color Missions

**DOI:** 10.3390/s20123377

**Published:** 2020-06-15

**Authors:** Dan Zhao, Lian Feng

**Affiliations:** 1 School of Environmental Science and Engineering, Southern University of Science and Technology, Shenzhen 518055, China; 11930928@mail.sustech.edu.cn; 2 Shenzhen Municipal Engineering Lab of Environmental IoT Technologies, Southern University of Science and Technology, Shenzhen 518055, China

**Keywords:** GOCI, geostationary, ocean color, MODISA, Chl-a, DPVOs, diurnal changes

## Abstract

The first geostationary ocean color satellite mission (geostationary ocean color imager, or GOCI) has provided eight hourly observations per day over the western Pacific region since June 2010. GOCI imagery has been widely used to track the short-term dynamics of coastal and inland waters. Few studies have been performed to comprehensively assess the advantages of GOCI images in obtaining valid observations and estimating diurnal changes within the water column. Using the entire mission dataset between 2011 and 2017, these knowledge gaps were filled by comparing the daily percentages of valid observations (DPVOs) between GOCI and MODIS Aqua (MODISA) and by examining the diurnal changes in Chl-a over the East China Sea. The mean DPVOs of GOCI was 152.6% over the clear open ocean, suggesting that a daily valid coverage could be expected with GOCI. The GOCI DPVOs were ~26 times greater than the MODISA DPVOs; this pronounced difference was caused by the combined effects of their different observational frequencies and the more conservative quality flag system for MODISA. Diurnal changes in the GOCI-derived Chl-a were also found, with generally higher Chl-a in the afternoon than the morning and pronounced heterogeneities in the temporal and spatial domains. However, whether such diurnal changes are due to the real dynamics of the oceanic waters or artifacts of the satellite retrievals remains to be determined. This study provides the first comprehensive quantification of the unparalleled advantages of geostationary ocean color missions over polar orbiters, and the results highlights the importance of geostationary ocean color missions in studying coastal and inland waters.

## 1. Introduction

Over the past few decades, various satellite ocean color instruments have been launched into space by many agencies, including the U.S. National Aeronautics and Space Administration (NASA), the National Oceanic and Atmospheric Administsaration (NOAA) and the European Space Agency (ESA) [1]. Most current and past missions are polar orbiters, such as the sea-viewing wide field-of-view sensor (SeaWiFS; 1997–2010), the moderate resolution imaging spectroradiometer (MODIS; 1999 to present for Terra and 2002 to present for Aqua), the visible infrared imaging radiometer suite (VIIRS; 2011 to present), the medium resolution imaging spectrometer (MERIS; 2002–2012) and the Sentinel-3 ocean and land color instrument (OLCI; 2016 to present). These instruments have provided valuable information for examining the surface processes of the oceans in both the biophysical domain and the biogeochemical domain and at regional to global spatial scales [2].

However, the aforementioned polar orbiters face a considerable challenge: there are sensitive to unfavorable observational conditions, including clouds, stray light and large solar/viewing angles; as a result, even with the acquisition of daily global data, valid ocean color observations are limited. For example, when the temporal resolution of MODIS Aqua (MODISA) is ~1 image/per day, its global mean probability to have one valid chlorophyll-a (Chl-a) retrieval per day is ~5% [3]. An even lower probability was found for data from the normalized florescence line height (nFLH; in mW cm^−2^ µm^−1^ sr^−1^) algorithm due to the relatively severe contamination of sun glint. Moreover, potential sampling bias could occur when infrequent sun-synchronous data are used to study the dynamics of coastal and inland waters, where the water optical properties exhibit evident short-term (e.g., diurnal to weekly) variability [4].

Geostationary satellite missions could overcome the scarcity of data by providing multiple observations per day. For example, the first geostationary ocean color imager (GOCI, data available since 2011), operated by the Korea Institute of Ocean Science & Technology, has demonstrated its unique contributions in capturing the diurnal changes of red tides, turbidity plumes, water quality and ocean surface current, etc. [5,6,7,8,9,10,11,12], given its temporal resolution of eight hourly observations per day. Although they have coarse spectral and spatial resolutions, geostationary meteorological satellites (such as the Himawari-8 from the Japan Meteorological Agency and the Geostationary Operational Environmental Satellite (GOES) from NOAA) boast high observational frequencies (tens of minutes), allowing them to detect, for example, the short-term dynamics of floating algae [13,14,15]. Ongoing and scheduled geostationary ocean color missions, including GOCI II (scheduled to launch in 2020) [16,17] and the geostationary coastal and air pollution events (GEO-CAPE) mission from NASA (currently cancelled due to budget cuts) [18,19], were also planned due to the pronounced improvement in their data coverage. Geostationary ocean color missions have also been discussed throughout the ocean color community in China; as a result, various prelaunch advances have been accomplished, including the development of a prototype instrument (http://www.chinabeidou.gov.cn/xinwen/2429.html) and the introduction of a vector radiative transfer model to account for the effects of the Earth’s curvature on Rayleigh scattering [20].

One of the most important tasks that must be performed before launching a new geostationary satellite is to determine the mission necessity, that is, determining what can be missed with low Earth orbit (LEO) instruments. The improved coverage of a geostationary mission can be indirectly inferred through statistics of cloud-free days using hourly cloud coverage observations from meteorological satellites (i.e., the GOES system) [19]. Indeed, more than one cloud-free observation is expected over the Intra-Americas Sea when geostationary satellite observations are available [19] given the mean daily cloud coverage of 72% for the global ocean [21]. However, such statistics are based on meteorological satellite data rather than real ocean color observations; hence, the extent to which the valid data coverage of ocean color retrievals can be improved through geostationary missions remains unknown. Note that “valid” here means that the data are not contaminated by the nonoptimal observational conditions mentioned above.

Real geostationary measurements from GOCI have been used to demonstrate its advantages over coastal oceans by tracking the diurnal changes in the sediment plumes of riverine estuaries [6,22], documenting the short-term spatial and vertical redistributions of harmful algal blooms [5,7,23], and detecting the continuous evolution of internal waves [24], etc. In the open ocean, however, the optical characteristics of the water column have commonly been considered diurnally invariant; this assumption has even been applied to determine the accuracy of the GOCI ocean color products [25]. In contrast, previous in situ investigations have demonstrated that the concentrations of water constituents and their optical properties vary considerably over a short period (daily or hourly) in clear oceanic waters [26,27,28]. Therefore, whether the optical features of the open ocean exhibit diurnal variations within GOCI observations remains unclear, and this is also the main focus of the current study.

Fortunately, the availability of hourly GOCI data starting from 2011 makes it possible not only to fully assess the advantages of geostationary missions over LEO satellites with regard to the coverage of valid data, but also to quantify the short-term variations in both coastal and open oceans. The current study was therefore designed with the following objectives:

1. Compare the daily percentages of valid observations (DPVOs) of Chl-a between MODISA and hourly and daily GOCI measurements and assess the diurnal changes in ocean color products at different locations;
2. Demonstrate how differences in satellite orbits, observational frequencies and data processing methods could impact the data coverage and ocean color measurements;
3. Discuss how the results of this study could be used to help both mission plan of future geostationary ocean color missions and associated algorithm development.

## 2. Data and Methods

### 2.1. Datasets and Preprocessing

**Figure 1:**
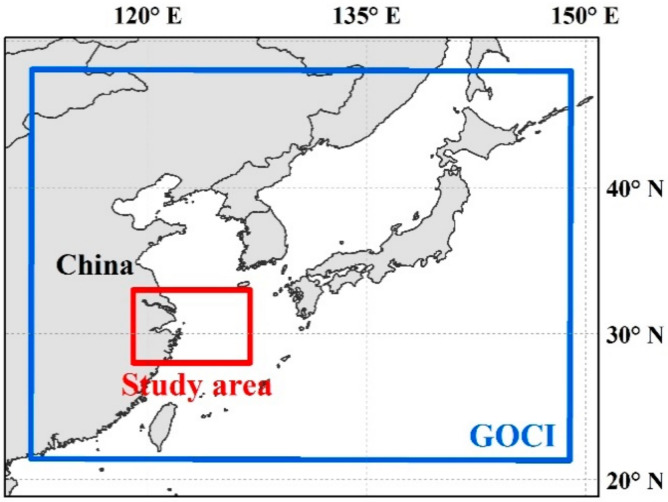
Location of the study area (red box), which is located to the east of China, covering inland water, coastal and offshore of the East China Sea within the coverage area of geostationary ocean color imager (GOCI) (blue box).

MODISA local area coverage (LAC) and GOCI data covering the Yangtze River Estuary and coastal oceans (119°–127° E, 28°–33° N), where the water types range from productive inland waters (e.g., Taihu Lake) to turbid coastal waters and then to clear oceanic waters, were selected in this study (Figure 1). The range of Chl-a concentration in the East China Sea is from <1 to >10 mg m^−3^ [29], and such values could be magnitudes higher in Taihu Lake [30]. Level-2 MODISA and GOCI products between April 2011 and December 2017 were downloaded from the NASA Goddard Space Flight Center (GSFC) (https://oceancolor.gsfc.nasa.gov); a total of 18,473 GOCI granules and 4440 MODISA granules were obtained in this 7-year time window, and all these images were used in the following analysis. The exclusion of data in recent years from this study was due to the decreased availability of GOCI data. The Level-2 products for both instruments were generated from the most recent preprocessing by the ocean biology processing group (OBPG), that is, R2018.0 for MODISA and R2014.0 for GOCI. Level-2 ocean color products is a suite of the geophysical parameter retrievals at the same resolution as the Level-1 data (1 km for MODISA and 500 m for GOCI), including the remote sensing reflectance (R_rs_), Chl-a, aerosol optical thickness, diffuse attenuation coefficient at 490 nm (K_490_), among others. A 32-bit quality flag (l2_flag) is also contained in each Level-2 file, which is used to indicate the quality of the ocean color products for each pixel. To facilitate further comparison between these two satellite missions, the GOCI and MODISA data were reprojected onto the same cylindrical equidistant (rectangular) projection with the same spatial resolution of 1 km using the latest SeaDAS software package (version 7.5).

Chl-a concentrations (in mg m^−3^) contained within the Level-2 product files were used in this study to examine the differences between MODIS and GOCI observations. Chl-a is the most important ocean color parameter because it represents the primary production and eutrophic status of water [31]. The Chl-a observations from GOCI were calculated using an empirical band ratio algorithm [32]. In contrast, the Chl-a observations from MODISA were derived using two algorithms: in the first, Chl-a of oligotrophic water (Chl-a < 0.15 mg m^−3^) was estimated using the Hu color index algorithm [33]; and in the second, the blue-to-green band ratio algorithm was applied to high-Chl-a waters [34]. And in between these values, the Hu’s and band ratio algorithm are blended using a weighted method (https://oceancolor.gsfc.nasa.gov/atbd/chlor_a/). The detailed Chl-a calculation procedures for GOCI and MODISA are as follows:

For GOCI:

(1) $$\log_{10}\left(\mathrm{Chla}\right)=a_{0}+\sum_{i=1}^{4}a_{i}{\left(\log_{10}\left(\frac{R_{\mathrm{rs}}\left(\lambda_{\mathrm{blue}}\right)}{R_{\mathrm{rs}}\left(555\right)}\right)\right)}^{i}.$$

For MODISA:

(2) $$\left\{\begin{array}{c} \mathrm{CI}=R_{\mathrm{rs}}\left(555\right)-\left[R_{\mathrm{rs}}\left(443\right)+\frac{555-443}{667-443}\times \left(R_{\mathrm{rs}}\left(667\right)-R_{\mathrm{rs}}\left(443\right)\right)\right]\;\\ \log_{10}\left(\mathrm{Chla}\right)=-0.4909+191.6590*\mathrm{CI}\;;\;\mathrm{Chla}<0.15\;\mathrm{mg}\;m^{-3}\;\\ \log_{10}\left(\mathrm{Chla}\right)=a_{0}+\sum_{i=1}^{4}a_{i}{\left(\log_{10}\left(\frac{R_{\mathrm{rs}}\left(\lambda_{\mathrm{blue}}\right)}{R_{\mathrm{rs}}\left(547\right)}\right)\right)}^{i}\;;\;\mathrm{Chla}\geq 0.2\;\mathrm{mg}\;m^{-3} \end{array}\right.,$$

where $R_{\mathrm{rs}}\left(\lambda_{\mathrm{blue}}\right)$ is the largest $R_{\mathrm{rs}}$ at blue wavelengths (443 and 489 nm for GOCI and 443 and 488 nm for MODISA); and the coefficients ($a_{0}$–$a_{4}$) are sensor-specific, which are 0.2515, −2.3798, 1.5823, −3.1747 and 0.0.3383 for GOCI and 0.2424, −2.7423, 1.8017, 0.0015 and −1.2280 for MODISA.

### 2.2. Estimation of the DPVOs

Valid Chl-a retrievals were first determined for each MODISA and GOCI image. By definition, “valid” means that a pixel is not associated with pre-defined quality control flag (e.g., l2_flag); this approach is adopted from the convention of the NASA OBPG employed to compose Level-3 data from Level-2 data [3,35]. Flags include the following: land mask, high sun glint, high calibrated radiance, atmospheric correction failure/warning, high satellite/solar zenith angle, stray light from clouds and land, cloud/ice mask, low water-leaving radiance, Chl-a algorithm failure/warning, navigation warning/failure, and maximum iteration exceeded.

The number of valid retrievals (Nv) during different time intervals (monthly, seasonal, annual and long-term mean values) was estimated by integrating the data (either MODISA or GOCI) within various time windows. Then, the DPVOs were estimated by normalizing Nv against the number of days during the examined periods, which could be expressed as follows:

(3) $$\mathrm{DPVO}=\frac{\mathrm{Nv}}{N\_\mathrm{days}}\times 100\%,$$

where N_days is the number of days during the examined periods. The mean DPVOs of the 8 hourly observations acquired by GOCI per day were first estimated to represent daily mean GOCI Chl-a, which was then utilized to compose monthly mean GOCI Chl-a. The long-term mean (LTM) DPVOs between April 2011 and December 2017 were then estimated as the mean DPVOs of the 81 monthly mean values, and the long-term seasonal mean (LTSM) datasets were represented as the means of the monthly data in the same season. The LTM and LTSM DPVO calculations were performed for both MODISA and GOCI. The same calculations were also conducted for GOCI data collected at 13:16 local time (denoted as GOCI_MODISA_) only, which constitutes the closet acquisition time to that of MODISA (~13:30 local time). These datasets were subsequently used to compare the capabilities of obtaining valid retrievals between GOCI_MODISA_ and MODISA given their similar observational frequencies (~1 image per day).

To quantify the influence of major contamination on DPVOs, we also estimated the daily percentage of cloud cover, high sunglint, atmospheric correction failure, high solar zenith angle, high sensor zenith angle, Chl-a algorithm failure, high radiance or saturated light and straylight for both MODISA, GOCI_MODISA_ and GOCI. The calculation procedures were similar to that for the DPVOs, where the numbers of these unfavorable conditions (as indicated by the l2_flags) were used to normalize against the number of days during the examined periods. Note that two or more unfavorable conditions could occur for one pixel and the summation of the daily percentage for all contamination factors could exceed 100%.

### 2.3. Analysis of Diurnal Changes in Chl-a

The LTM and LTSM composites of Chl-a were estimated using a similar method to those of the DPVOs, and the calculations were processed for both MODISA and GOCI. The relative differences between the LTM and LTSM Chl-a were calculated among MODISA, GOCI_MODISA_ and GOCI to assess the disparities in the ocean color products between different missions and between different observational frequencies.

To examine the diurnal changes in Chl-a, the ratio between each hourly GOCI Chl-a and the mean Chl-a of 8 hourly GOCI observations within the same day (denoted as DR_chla_) was also calculated. For each location in the study region, the DR_chla_ calculation was only conducted for the dates when 8 valid hourly GOCI Chl-a retrievals within a day are available. Seasonal and annual mean DR_chla_ for each year between 2011 and 2017 were estimated and then used to examine the seasonal and long-term patterns of the diurnal changes. To determine whether the diurnal changes are statistically meaningful (i.e., within the entire variability range for each hour), the standard deviations of all annual (seasonal) mean DR_chla_ from 2011–2017 were estimated for each hourly GOCI observation.

## 3. Results and Discussion

### 3.1. Comparison of DPVOs between Different Satellite Missions and Observational Frequencies

**Figure 2:**
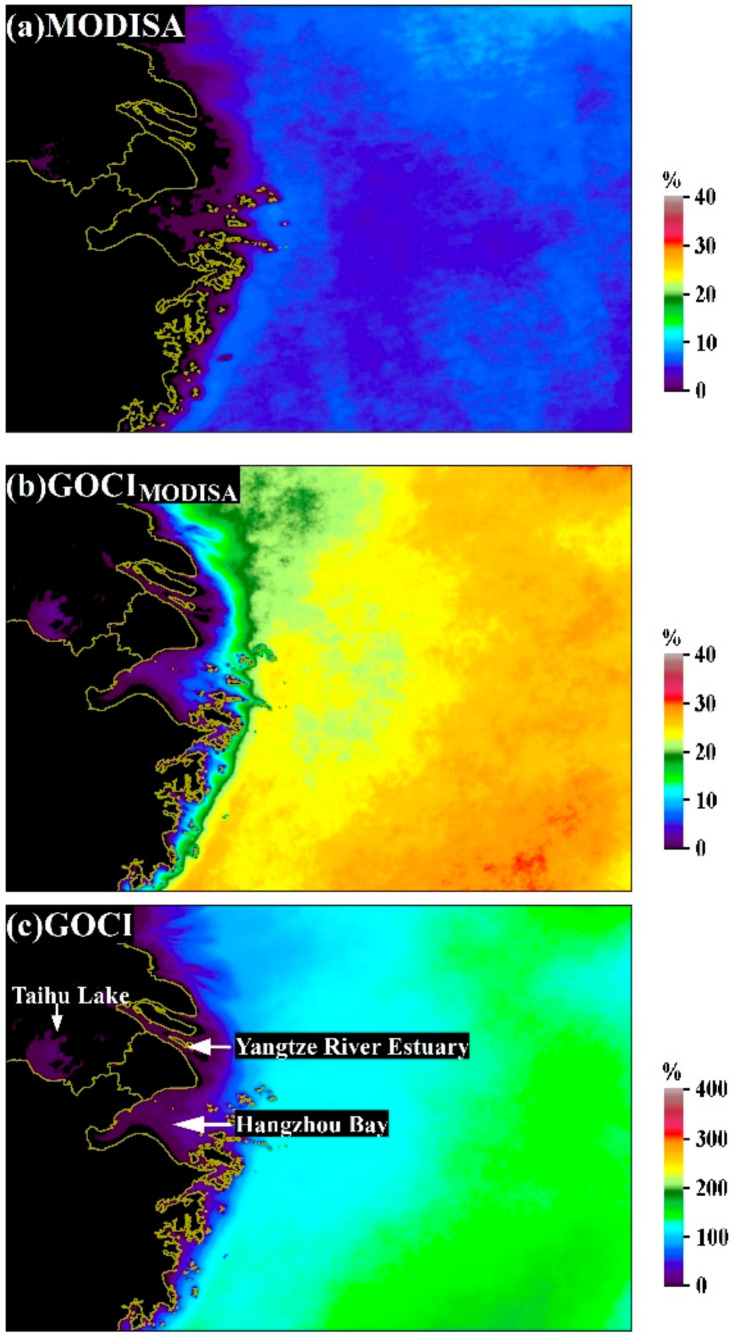
Long-term mean daily percentages of valid observations (DPVOs) of Chl-a between 2011 and 2017 for (**a**) MODIS Aqua (MODISA), (**b**) GOCI_MODISA_ and (**c**) GOCI, which were estimated as the mean values of 81 monthly DPVOs between April 2011 and December 2017.

The DPVOs for Chl-a during the entire examined period (2011–2017) are shown in Figure 2. The DPVOs were generally larger in the open ocean than in the coastal areas for both MODISA and GOCI (Figure 2a,c). The mean DPVO for MODISA Chl-a was 5.2% ± 0.6% (mean ± standard deviation), which is more than one order of magnitude less than that of GOCI (112.0% ± 0.1%). Valid ocean color products were not available for certain regions in Taihu Lake and some coastal oceans (such as the Yangtze River Estuary and Hangzhou Bay) with MODISA measurements, and GOCI measurements were able to fill the data gap.

**Figure 3:**
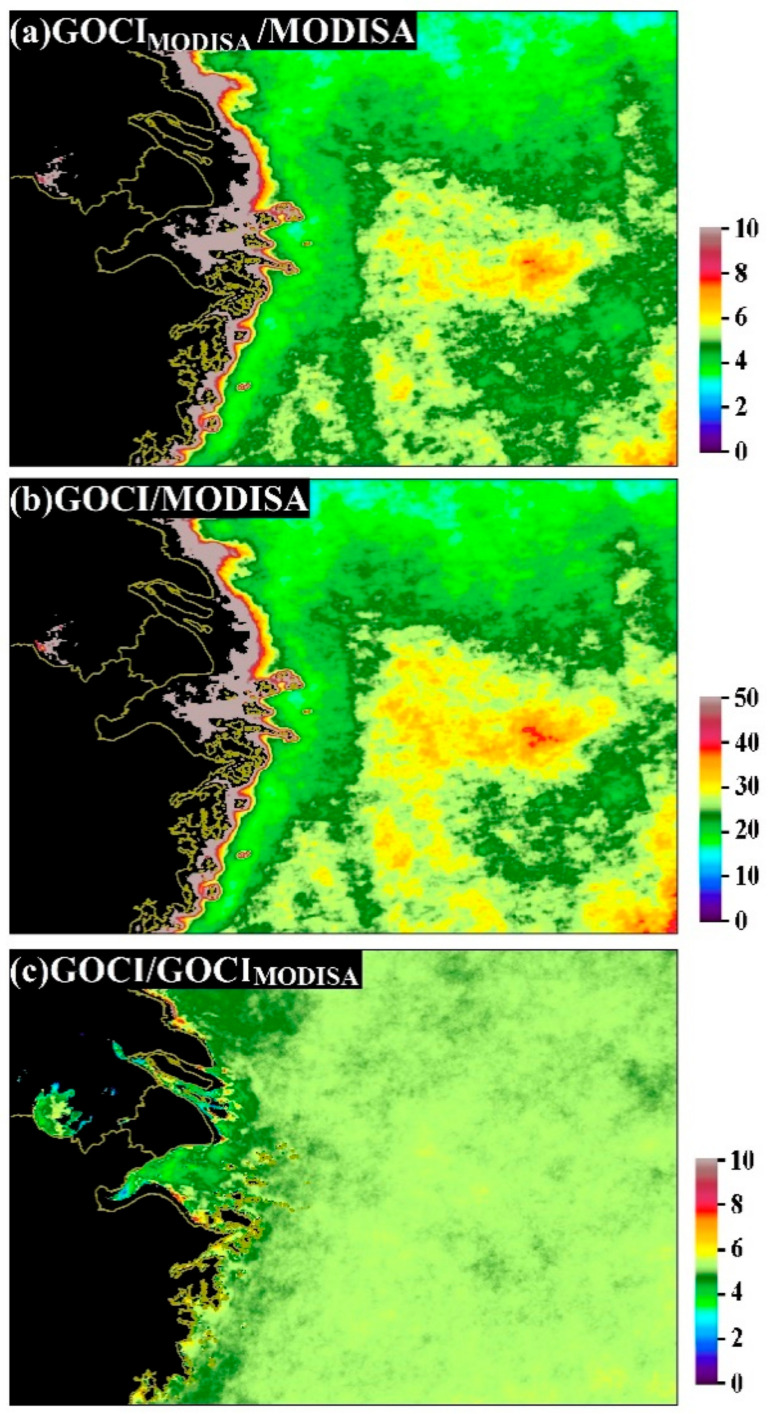
Ratios of the long-term mean DPVOs of Chl-a between 2011 and 2017 for (**a**) GOCI_MODISA_ and MODISA, (**b**) GOCI and MODISA and (**c**) GOCI and GOCI_MODISA_.

The prevailing advantage of GOCI with regard to providing complete data coverage was also revealed through the ratio of DPVOs between GOCI and MODISA (see Figure 3b); the DPVO of GOCI at most locations was at least 10 times that of MODISA (green to reddish colors in Figure 3b). The mean DPVO ratio of the entire region was 25.05 ± 47.1, but significantly higher ratios (>50, reddish to grayish colors) were found in some inshore regions, and relatively high ratios (>30, yellowish to reddish colors) were observed in the southeastern extent of the study region.

With similar temporal resolutions, GOCI_MODISA_ showed evidently higher DPVOs than MODISA, and the LTM Chl-a DPVO for GOCI_MODISA_ was 22.0% ± 2.0% (see Figure 2b), which is >4 times that for MODISA. The distribution of the ratio between DPVOs of GOCI_MODISA_ and MODISA is illustrated in Figure 3a, where the spatial patterns are almost identical to the ratio of DPVOs between GOCI and MODISA (i.e., the highest values were found in the inshore areas). The mean ratio of DPVOs between GOCI_MODISA_ and MODISA was 4.82 ± 16.75 for the entire study region, meaning that the average capability of GOCI_MODISA_ in obtaining valid ocean color retrievals was 4.82 times that of MODISA given their similar observational frequencies. The mean ratio between DPVOs of GOCI and GOCI_MODISA_ for the study region was 5.22 ± 0.89 (see Figure 3c), which is smaller than the difference in their temporal resolutions (1 vs. 8 hourly observations per day).

**Figure 4:**
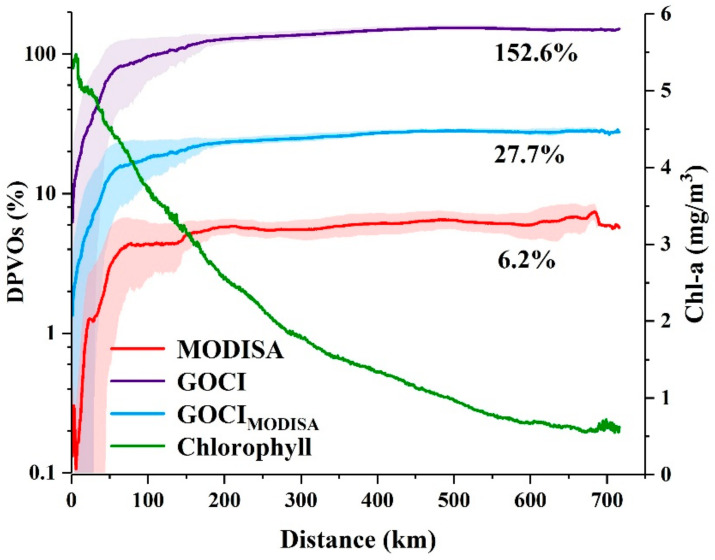
Long-term mean DPVOs of Chl-a between 2011 and 2017 as a function of the distance from the coastline for MODISA, GOCI_MODISA_ and GOCI. The standard deviations are shaded in the corresponding color with the mean values and plotted as the long-term mean Chl-a.

The DPVOs of Chl-a are plotted as a function of the distance from the coastline in Figure 4, where the values on the curves represent the mean DPVO of all pixels (shown in Figure 2) at a given distance to the coastline, and the associated standard deviation is shaded in the corresponding color along each curve. Small mean DPVOs were found near the shoreline, where the probability of obtaining one valid retrieval per day was < 10%, even with 8 daily observations provided by GOCI. In contrast, DPVOs of GOCI_MODISA_ and MODISA were orders of magnitude smaller than those of GOCI. For all three types of calculations, DPVOs increased steadily with increasing distance from coastline and then reached stable levels at a distance of approximately 150 km from the shoreline. The chances of acquiring valid data in the open ocean (distance from coastline > 150 km) were approximately one order of magnitude larger than those along the coastline regardless of the mission and statistical method. Numerically, the mean DPVO in clear oceanic waters was 152.6% for GOCI, indicating that daily valid ocean color measurements could be expected with an observational frequency of 8 images per day. The mean DPVO for GOCI_MODISA_ was estimated as 27.7%, accounting for 18.2% of that for GOCI. In contrast, MODISA displayed a mean DPVO of 6.2% in the offshore regions, which is consistent with the calculations of Feng and Hu [3]. This finding also suggests that the temporal coverage of valid observations (i.e., 6.2%) for MODISA is far less than its satellite revisit frequency (~1 image per day).

**Figure 5:**
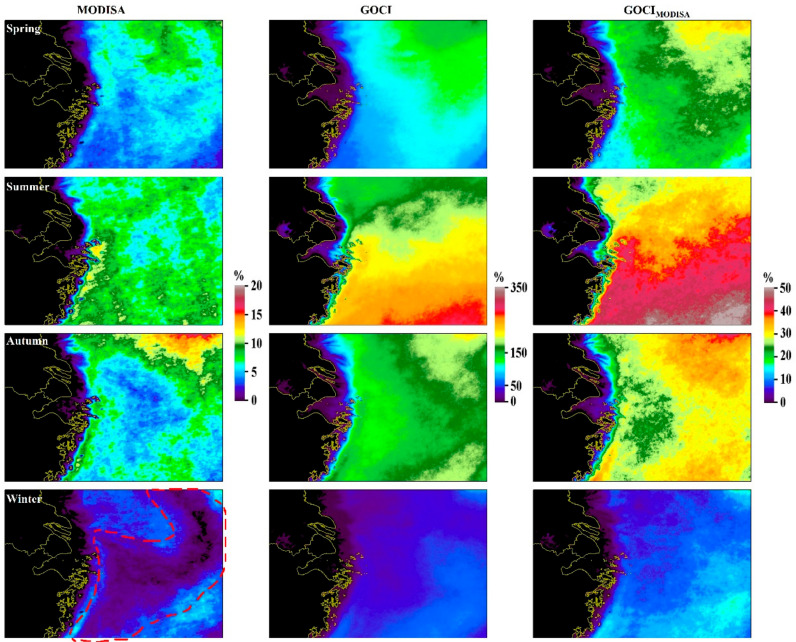
Long-term seasonal mean DPVOs for Chl-a between 2011 and 2017 for MODISA, GOCI_MODISA_ and GOCI. A tongue-shaped region of low DPVOs for MODISA is encircled in a red dashed line.

The significant interannual dynamics of DPVOs are revealed through the LTSM values in Figure 5. Compared with the apparent ocean-to-land gradient in the LTM DPVOs (see Figure 2), the LTSM DPVOs demonstrated greater spatial heterogeneities in all four seasons. For example, while the northeastern part of the study region showed higher DPVOs in spring, the highest DPVOs were observed in the southeast in summer. Indeed, a large tongue-shaped region of low DPVOs was clearly delineated from the southwest to the northeast in winter for MODISA (area encircled by a red dashed line in Figure 5), while this pattern was not found for either GOCI or GOCI_MODISA_. In general, more valid ocean color data were expected in summer and autumn than in spring and winter, which is due to the less cloudy days in warmer seasons [21]. The smallest possibilities to obtain valid data occurred in winter for both instruments (2.1% ± 0.1% for MODISA and 40.7% ± 5.1% for GOCI), where the DPVOs are less than half of those in the other seasons.

### 3.2. Diurnal Changes in GOCI-Derived Chl-a

**Figure 6:**
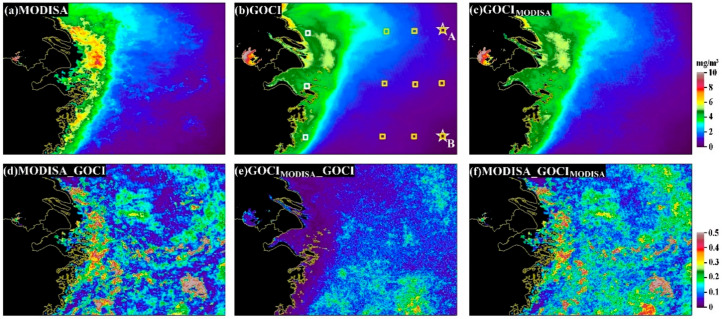
(**a**–**c**) Long-term mean Chl-a between 2011 and 2017 for MODISA, GOCI_MODISA_ and GOCI, as well as their absolute relative differences (**d**–**f**). The stars in (**b**) indicate the locations for points A and B used to generate Figure 7, and the white/yellow squares indicate the locations for inshore/offshore calculations for Table 2.

The LTM Chl-a estimates for MODISA, GOCI_MODISA_ and GOCI and their relative differences are shown in Figure 6. The Chl-a products shared very similar spatial patterns among the three types of calculations; larger Chl-a values were found in western inland and coastal waters, and they decreased gradually toward the open ocean in the east. Such a spatial gradient was also illustrated in Figure 4. In contrast, out-of-phase spatial patterns were identified for the relative differences between GOCI_MODISA_ and GOCI (i.e., their relative differences were evidently lower in inshore regions than in offshore regions).

**Figure 7:**
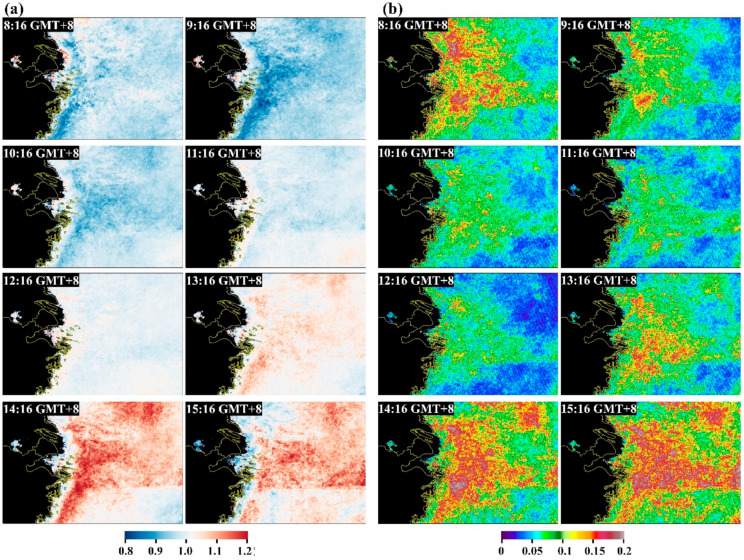
(**a**) Long-term mean ratio between each hourly GOCI Chl-a and the mean Chl-a of 8 hourly GOCI observations (i.e., DR_chla_), demonstrating the diurnal changes in the GOCI-derived Chl-a; (**b**) standard deviations of all annual mean DR_chla_ from 2011–2017 for each hourly GOCI observation, showing the interannual variations of the diurnal changes.

**Figure 8:**
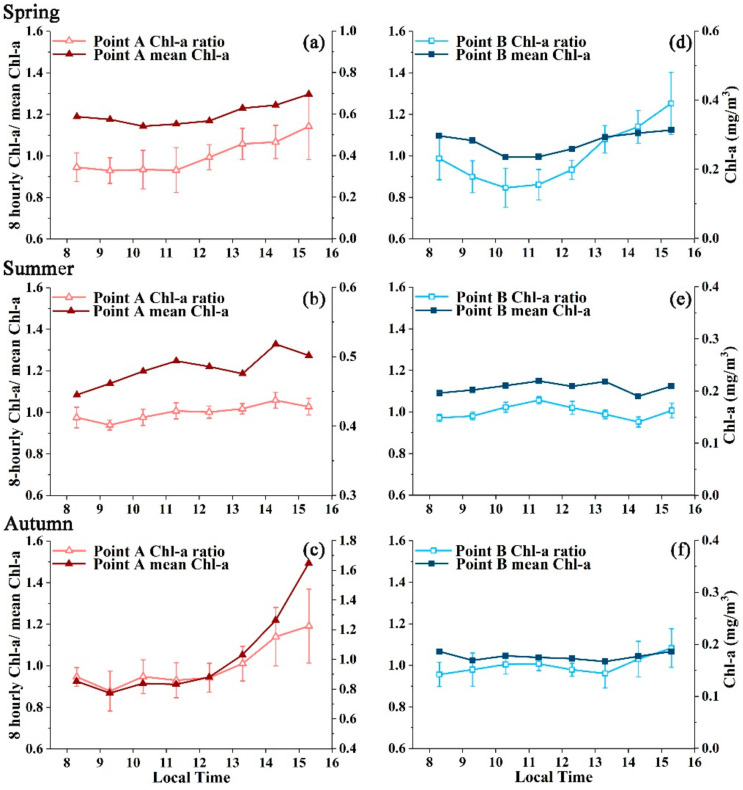
Long-term mean ratio between each hourly GOCI Chl-a and the mean Chl-a of 8 hourly GOCI observations (DR_chla_) for point A (panel **a**–**c**) and point B (panel **d**–**f**) in spring, summer and autumn (the locations of A and B are annotated in Figure 6b). The diurnal variations in the Chl-a concentrations are clearly revealed through these figures. Error bars indicate the standard deviations of all seasonal mean DR_chla_ during 2011–2017 for each hourly GOCI observation.

The diurnal changes in Chl-a are revealed through the LTM DR_chla_ in Figure 7a. In general, the afternoon data displayed large ratios (>1) throughout most of the study region, indicating higher Chl-a retrievals in the afternoon. Another interesting finding is that these high afternoon ratios generally occurred in open ocean rather than in inland and coastal waters. Conversely, the ratio in the morning was generally smaller than that in the afternoon (<1), and high morning ratios were found mostly in productive coastal and inland oceans, thereby opposing the afternoon patterns. Substantial spatial heterogeneities could also be revealed through the significant differences in DR_chla_ for two specific points (see Figure 8 and Table 1), where the diurnal changes in point A appeared to differ from that of point B despite the two points presenting similar Chl-a magnitudes and distance to coastline.

Significant seasonal fluctuations are superimposed on the pronounced spatial dynamics of the diurnal dynamics in Chl-a (see Figure 8). The Chl-a concentrations showed the smallest diurnal changes at points A and B in summer as demonstrated by the close proximity to 1 for the DR_chla_ of all 8 hourly GOCI observations. The most pronounced sub-daily changes in LTSM DR_chla_ for point A occurred in autumn, with the values ranging from 0.88 at 9:16 am to 1.19 at 15:16pm (see Table 1). The largest changes of LTSM DR_chla_ for point B were found in spring, with the values ranging from 0.85 at 10:16 am to 1.26 at 15:16 pm. Note that due to the strict data selection criteria (i.e., 8 hourly Chl-a retrievals are available within a day), winter data were not available for these two points to conduct DR_chla_ calculations.

The standard deviations of annual DR_chla_ from 2011–2017 for the entire study region was estimated in Figure 7b. Clearly, the standard deviations are below the diurnal changes of DR_chla_, suggesting that the sub-daily changes in GOCI-observed Ch-a could be statistically meaningful. Indeed, such patterns could be also found at seasonal scales (see error bars associated with each hourly observation in Figure 8 and numbers in Table 1), where the interannual variations (i.e., standard deviations) were smaller than the magnitude of the diurnal changes.

### 3.3. Factors Leading to Discrepancies in the DPVOs

The fact that the DPVOs of GOCI were higher than those of GOCI_MODISA_ and MODISA was apparently due to differences in their observational frequencies; GOCI provides 8 hourly observations a day, while the other two offer ~1 image per day. The mean DPVO for GOCI_MODISA_ was 25% for clear open ocean, which is slightly less than the clear sky probability derived from the cloud coverage dataset (the mean cloud cover fraction over this region is approximately 70%) (see Figure 1 in King, Platnick, Menzel, Ackerman and Hubanks [21]), suggesting that the presence of clouds is the most crucial factor impacting the DPVOs. Indeed, remarkably higher cloud coverage was found for GOCI_MODISA_ than for MODISA, which was likely caused by different cloud masking scheme between these two instruments since they had the same observational frequency and a similar acquisition time. Nevertheless, the GOCI mission even yielded much higher DPVOs, which could be associated with the differences in their processing schemes, satellite geometries and instrument designations.

The quality control flag system of MODISA used to generate Level-3 products appears more conservative than that of GOCI. Under cloud-free conditions, “straylight”-flagged pixels were approximately an order of magnitude larger than the DPVOs for both inshore and offshore regions in the study area (see Table 2). Such magnitudes were even greater than the global mean values, where a “straylight” flag could result in a data coverage reduction of >50% among the total cloud-free data for MODISA [36]. In contrast, the “straylight” flag was not applied for GOCI data (https://ebcrpa.jamstec.go.jp/egcr/e/AWOC2015/P5_H_Yang.pdf), resulting in a significant increase in data coverage. The purpose of applying the stray light flag near clouds and land is to eliminate the impacts of adjacency effects from bright targets on neighboring water pixels [36,37], where a 7 × 5-pixel window would be masked if the central pixel is identified as cloud or land. Nevertheless, the current stray light mask could be relaxed to a 3 × 3 pixel window without sacrificing data quality, and an increase in valid data coverage of ~40% could be expected through this mask relaxing scheme [38]. However, the exclusion of stray light flag from GOCI data should be fully investigated, although the processes with and without a 7 × 5-pixel window mask would not generate significant differences in diurnal changes revealed in this study (results not shown here).

Furthermore, when the sensor zenith angle exceeds a threshold (60°), a “HISATZEN” flag of the l2_flag will be associated with the pixel [35], and the data will be excluded when composing Level-3 data. As MODISA is a polar orbiter with a swath width of 2330 km, the sensor zenith angle could be larger than the threshold at scan edges; consequently, the data would be discarded as invalid. Numerically, such a reduction represents > 15% of the total data coverage of MODIS (see Table 2). In contrast, the sensor zenith angles for the geostationary orbiter GOCI should always be less than such a threshold because of the high altitude of satellite, and thus, no data would be excluded due to the large sensor zenith angle.

Pixels flagged as “HILT” (very high or saturated observed radiance) accounted for another substantial data loss source in MODISA, and such pixels appeared more in coastal high reflective regions than offshore oceans (Table 2) because the ocean bands of MODISA are prone to be saturated due to their high sensitivity designation and small dynamic ranges (Hu et al., 2012). In contrast, the GOCI data over the entire study region never suffer from this issue.

**Figure 9:**
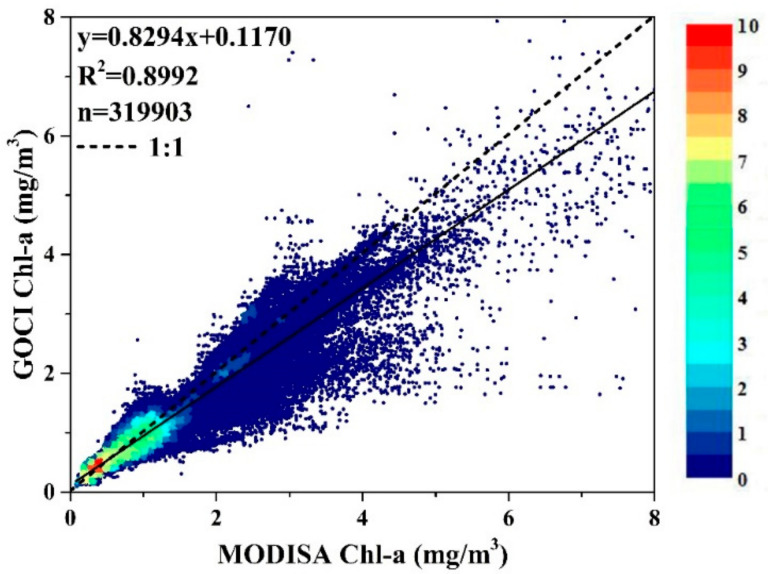
Density plot showing the correlations between concurrent GOCI (i.e., GOCI_MODISA_) and MODISA Chl-a estimates. Legend represents the frequency of the data values (dimensionless).

The differences in the LTM Chl-a between MODISA and GOCI_MODISA_ could have been generated by a combined effect of the disparities in the retrieval algorithms (GOCI uses a band ratio algorithm, while MODISA employs merged band ratio and band difference algorithms), satellite geometries, band configurations (i.e., wavelengths) and water property variations associated with small temporal differences. To examine the correlations between the GOCI_MODISA_ and MODISA Chl-a, concurrent match-ups from the two satellites are plotted against each other in Figure 9. Although significant correlations were found between the two independent observations (*R^2^* = 0.9, *p* < 0.01), a substantial amount of data points were distributed far away from both the 1:1 line and the regression line (particularly for large Chl-a values). Statistically, the MODISA Chl-a values were larger than the GOCI_MODISA_ Chl-a values, agreeing well with the findings posted by the OBPG (https://oceancolor.gsfc.nasa.gov/reprocessing/r2014/goci/). The discrepancies between the two types of Chl-a products could be associated with various differences between GOCI and MODISA, including band configurations, spectral responses, signal-to-noise ratios (SNRs) and atmospheric correction residual errors, etc.

### 3.4. Interpretation of the Diurnal Changes in GOCI Chl-a Retrievals

The long-term GOCI observations revealed rapid subdiurnal variations in Chl-a concentrations in oceanic waters. Such dynamics could be significant in coastal oceans (particularly in spring and autumn, see Figure 7) and could be partially linked with the short-term dynamics of water optical properties. Specifically, the optical properties of these productive waters could be easily modulated by various physical and biogeochemical processes on ocean-land interfaces triggered by natural and/or anthropogenic forces (e.g., tides, winds and waves, river discharge and point and nonpoint pollution). The most interesting finding of this study is the comparable or even more pronounced diurnal changes in offshore region. Such results appear inconsistent with the conventional understanding of the physical and biogeochemical characteristics of the open ocean, where changes in water constituents and the associated optical properties are considered negligible over the course of a day. For example, water columns in an open ocean region were assumed to be diurnally invariant by Concha, Mannino, Franz and Kim [25], and they used this assumption to gauge the uncertainties in GOCI ocean color products.

Whether the diurnal changes in GOCI Chl-a represent real variations in the water column or the artifacts of satellite observations (and the associated processing procedures) remains to be answered. To address this question, concurrent hourly in situ measurements are required to assess the fidelity of the hourly GOCI Chl-a retrievals. Unfortunately, such field data are currently unavailable, prohibiting direct validations of hourly GOCI ocean color products. Nevertheless, the short-term variability of GOCI Chl-a in the open ocean could be attributed to the following factors.

The first factor is real changes in Chl-a concentrations in oceanic waters, which could also possibly occur within a short time period. For example, Neveux et al. [26] employed in situ measurements from the equatorial Pacific and showed that the growth and grazing rates of the phytoplankton pigments (including Chl-a, Chl-b, Chl-c and total Chl-a) were imbalanced over the course of a day, leading to considerable changes in the phytoplankton community structure and biomass in the upper layer with depths of 0–30 m (the changes reached up to 28% for Chl-a). Significant diel variations of zooplankton were also revealed by Le Borgne and Rodier [39] using data from two equatorial time series stations in the Pacific, where even the variations between zooplankton communities with different structures differed. Therefore, the short-term growth and mortality of pigments could potentially lead to considerable Chl-a dynamics in the surface oceans, as observed by the hourly GOCI images.

Diurnal changes in the mass-specific absorption coefficient (denoted as a*) [40,41] could serve as another important factor. The responses of phytoplankton to different environments (temperature, solar radiation, etc.) could lead to substantial variations in a* over a short period. A study by Mercado, Ramírez, Cortés, Sebastián, Reul and Bautista [28] in the Mediterranean Sea indicated that the accumulation effects of photoprotective pigments (such as carotenoids) could be expected in the afternoon with an increase in incident irradiance, leading to an enhancement in a* at blue bands. Nevertheless, a combined decrease of Chl-a has been found with the increase of a* in their study. Further, Fujiki and Taguchi [42] also found significant photoprotective pigments associated with an increase in a* at 440 nm with increasing irradiance, and demonstrated that photoacclimation effects were independent of the growth and mortality of phytoplankton in the upper mixed layer [26]. Consequently, the reduced reflectance at the blue band could lead to overestimation of Chl-a retrievals, as satellite Chl-a values are negatively proportional to the blue/green reflection ratio [33,34]. Such photoacclimation processes agreed well with the diurnal patterns throughout the study region, where GOCI Chl-a retrievals appeared higher in the afternoon than in the morning (Figure 7 and Figure 8). In addition to the changes in pigment compositions, the packaging effects of pigment cells could also lead to variations in a* [27]. However, due to the absence of in situ observations of diurnal a* and Chl-a in this region, the determination of how the photoacclimation and packaging effects could impact satellite Chl-a retrievals requires further evidences.

Imperfections of data processing methods, including the lack of consideration for the Earth’s curvature, the bidirectional properties of remote sensing reflectance (R_rs_) and the strong polarization effects when conducting atmospheric corrections [20,43], could lead to artifacts in the derived R_rs_ and thus the GOCI Chl-a retrievals. As simulated by He et al. [20], the impacts of the Earth’s curvature and polarization could lead to errors reaching up to 12% at blue wavelengths when the solar zenith is high (e.g., 85°). While such artifacts may potentially be present in the observed Chl-a dynamics, their roles in modulating these significant diurnal changes should be small because angular-dependent uncertainties do not cause the heterogeneous spatial/temporal patterns of the short-term variability (see Figure 7). Additionally, the impacts associated with the solar zenith angle appeared to be negligible in summer and autumn, when the solar zenith is small (even in the morning), while significant daily variations in Chl-a could also be found in certain areas (see Figure 8 and Table 1), suggesting that other factors play roles in controlling the diurnal changes in Chl-a.

Artifacts of remote sensing instruments may also contribute to the diurnal changes in final ocean color estimations. For example, the evident radiometric inconsistency between the adjacent slots of GOCI images or the interslot radiometric discrepancy (ISRD) could lead to radiometric anomalies and thus obvious discontinuous features in ocean color retrievals across slot boundaries. Although an attempt has been conducted to correct the effects of the ISRD [44], extensive validations are required to assess the robustness of the correction method before its implementation on subsequent data reprocessing. Another sensor-related issue is the phenomenon of diurnal changes in the SNR resulting from the variability of the incoming radiance. Although the SNR of GOCI at typical radiance is even higher than that of SeaWiFS [45], the SNR could be much smaller in the morning than in the afternoon due to an evident reduction in solar irradiance, influencing the detectability of Chl-a values. Similar to the issues related to the Earth’s curvature, such artifacts were not supposed to cause the significant diurnal changes in Chl-a observed in summer and autumn when the solar zenith angle was small, even at the acquisition time of the first daily GOCI image (8:16 local time).

The diurnal changes in Chl-a also demonstrated considerable spatial inhomogeneity across the study region. These spatial patterns may be attributed to the discrepancies in the species compositions and distributions of phytoplankton and thus the optical characteristics of the water column within this region [46]. Nevertheless, comprehensive field measurements are required to validate this assumption.

### 3.5. Implications for Future Geostationary Ocean Color Missions

**Figure 10:**
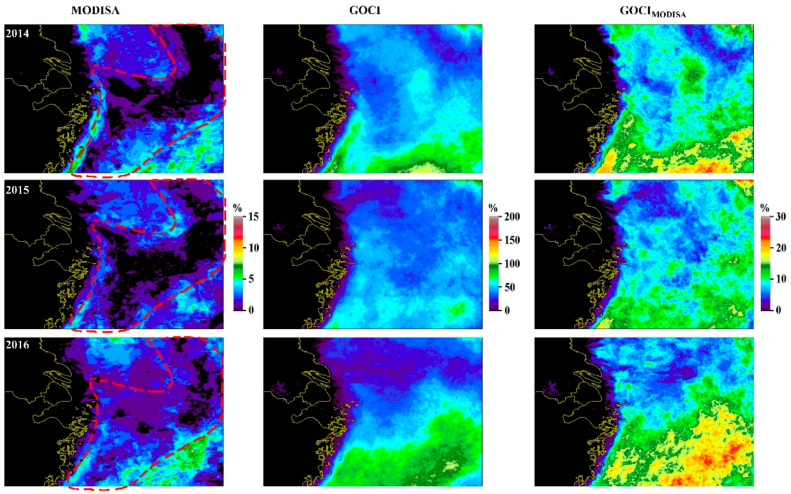
Mean DPVOs in winter between 2014 and 2016 for MODISA, GOCI_MODISA_ and GOCI. Tongue-shaped region of low DPVOs (red circled) was consistently found in MODISA, whereas data gaps were not expected with either GOCI_MODISA_ or GOCI. In fact, such phenomena have reoccurred every year between 2011 and 2017.

Significant diurnal changes in Chl-a were revealed through frequent GOCI observations (especially in the open oceans), and the change patterns diverged rapidly among the four seasons. Indeed, the results were similar to those reported by Arnone, Vandermeulen, Soto, Ladner, Ondrusek and Yang [4] using hourly overlapping VIIRS and in situ data. LEO instruments (such as MODISA and VIIRS) can capture the status of the ocean at only one specific moment and the associated trends/changes, as presented by numerous pioneering studies [47,48], may not be able to represent real transitions in the properties of the ocean; rather, they can represent only the changes for that certain moment. In contrast, the measurements from geostationary satellites (such as GOCI) could be more reliable for characterizing the processes corresponding to ocean optics given the ability to provide multiple observations within a day. Taking an extreme case as an example, as shown in Figure 5, a tongue-shaped region of low DPVOs was found in the MODISA LTSM DPVOs in winter (the DPVOs were less than 1% or even zero), meaning that rare or even no ocean color observations were available. However, such an observational blind spot could be complemented with GOCI. In fact, this low-DPVO feature reoccurred in the MODISA data for every year within the period of observation (see Figure 10 for the results of 2014–2016), suggesting the potential reoccurrence of a relatively stable physical process in this region. Indeed, such patterns agree well with the Zhe-Min coastal current, which has been suggested to be more severe in winter than in the other seasons [46,49], resulting in higher chances of cloud cover (see Table 2). However, the ocean color dynamics due to this process would be missed with MODISA due to the absence of valid measurements (e.g., most areas in this region showed zero DPVOs). In contrast, seamless 8 hourly composites of LTSM Chl-a images could be obtained with both GOCI and GOCI_MODISA_ in any single year in this area, making it possible to track changes in water masses and thus better understand this specific physical process.

The daily variations in Chl-a within inland lakes and coastal oceans were less evident than those within the open oceans in some seasons, but this does not mean that the diurnal dynamics of the optical properties of water are insignificant in these regions. Instead, this may be due to two reasons: (1) The DR_chla_ and standard deviations used here (Figure 7) represent the relative dynamics of Chl-a within the water column over the course of a day, and the variability of small Chl-a values in the offshore region could often lead to greater relative changes. The opposite is true for the coastal oceans, where the background Chl-a value is much higher than that for the open ocean (see Figure 4 and Figure 6). (2) The insufficient number of valid Chl-a retrievals in these regions, where effective atmospheric correction (AC) procedures are difficult to implement because of extreme turbidity-enhanced signals in the AC bands (i.e., near-infrared), constitutes a limitation [50,51,52]. Indeed, geostationary observations boast unique advantages in studying coastal and inland waters, where the short-term dynamics of optical properties should be remarkable due to various factors, including tide forcing, winds and waves, surface runoff and diurnal vertical migrations of algae [5,6,7].

Geostationary observations have demonstrated fundamental advantages in studying ocean Chl-a changes compared with polar orbiters, as indicated by the pronounced increase in data coverage and the capability to track diurnal changes. While such advantages are not limited to only Chl-a, other ocean color products (such as R_rs_ (in sr^−1^) and diffuse attenuation coefficients or K_d_ (in m^−1^)) also show heterogeneities in both spatial and temporal patterns, which are similar to those of Chl-a (results not shown here). Therefore, the requirements of geostationary ocean color missions are highlighted herein to capture diurnally unstable features in the open oceans and in coastal and inland productive waters.

The current Chl-a algorithm employed by both GOCI and MODIS represents the total absorption (a_T_) of the water column at blue wavelengths [34]. Therefore, any changes in a_T_ within a day caused either by real changes in Chl-a or by variations in a* (as discussed above) could be reflected as diurnal changes in satellite Chl-a values. Therefore, future efforts are required to develop a semianalytical Chl-a algorithm for geostationary satellite missions [32,53] to account for the subdaily variations in a* within the water column. Additionally, compared with the 3-h time window currently recommended by NASA, a 1-h time window is recommended to define “concurrency” between in situ and satellite observations when conducting validation efforts for ocean color products from geostationary missions [54].

## 4. Conclusions

The observational capabilities of the first geostationary ocean color mission (GOCI) were comprehensively assessed for the first time in terms of the number of valid observations and the diurnal changes in Chl-a using long-term data between 2011 and 2017. The DPVOs of GOCI were compared with those of MODISA and GOCI_MODISA_ to quantify the number of valid observations between different satellite missions and observational frequencies. The fact that the DPVOs of GOCI were higher than those of GOCI_MODISA_ and MODISA was due to the differences in their observational frequencies. Furthermore, cloud coverage and different processing schemes of quality control flag system are crucial factors impacting the DPVOs. The diurnal changes in Chl-a and the corresponding seasonality were analyzed using the hourly observations provided by GOCI, which could be the real variation of Chl-a concentrations and may also be affected by the biophysical processes in oceanic waters and the imperfections of remote sensing processing methods, such as photoacclimation effects, the lack of consideration for the earth’s curvature, artifacts of remote sensing instruments, etc. While additional datasets are required to ascertain the validity of the observed diurnal Chl-a fluctuations. The results demonstrated that geostationary satellite missions could not only provide a daily valid data coverage within the footprint, but also reveal the significant diurnal variability of the water column for both coastal waters and open oceans. The quantified advantages of geostationary satellites over LEO satellites further highlight the requirements for geostationary ocean color missions in the future and the discussion regarding the diurnal variability of Chl-a stresses the need for renewed efforts to develop an improved algorithm and to validate geostationary satellites.

## Figures and Tables

**Table 1:** Long-term seasonal mean ratio between each hourly GOCI Chl-a and the mean Chl-a of 8 hourly GOCI observations (DR_chla_). Long term seasonal mean Chl-a and the standard deviations of seasonal mean DR_chla_ from 2011–2017 are also listed for each hourly GOCI observation.

Local Time	Point A									Point B								
	Spring			Summer			Autumn			Spring			Summer			Autumn		
	Chl-a Ratio	Ratio Std	Mean Chl-a (mg/m^3^)	Chl-a Ratio	Ratio Std	Mean Chl-a (mg/m^3^)	Chl-a Ratio	Ratio Std	Mean Chl-a (mg/m^3^)	Chl-a Ratio	Ratio Std	Mean Chl-a (mg/m^3^)	Chl-a Ratio	Ratio Std	Mean Chl-a (mg/m^3^)	Chl-a Ratio	Ratio Std	Mean Chl-a (mg/m^3^)
8:16	0.95	0.07	0.59	0.98	0.05	0.45	0.95	0.05	0.85	0.99	0.10	0.30	0.97	0.02	0.20	0.96	0.06	0.19
9:16	0.93	0.06	0.57	0.94	0.02	0.46	0.88	0.10	0.77	0.90	0.08	0.28	0.98	0.02	0.20	0.98	0.08	0.17
10:16	0.93	0.09	0.54	0.98	0.04	0.48	0.95	0.08	0.84	0.85	0.09	0.24	1.02	0.02	0.21	1.00	0.05	0.18
11:16	0.93	0.11	0.55	1.01	0.04	0.49	0.93	0.08	0.83	0.86	0.07	0.24	1.06	0.02	0.22	1.01	0.03	0.17
12:16	0.99	0.06	0.57	1.00	0.03	0.49	0.94	0.07	0.88	0.93	0.05	0.26	1.02	0.03	0.21	0.98	0.03	0.17
13:16	1.06	0.08	0.63	1.02	0.02	0.48	1.01	0.08	1.03	1.08	0.07	0.29	0.99	0.02	0.22	0.96	0.07	0.17
14:16	1.07	0.08	0.64	1.06	0.04	0.52	1.14	0.14	1.26	1.14	0.08	0.30	0.95	0.02	0.19	1.03	0.09	0.18
15:16	1.14	0.16	0.70	1.03	0.04	0.50	1.19	0.18	1.65	1.25	0.15	0.31	1.01	0.04	0.21	1.08	0.09	0.19

**Table 2:** Mean daily percentages of various factors that could impact the DPVOs for MODISA, GOCIMODISA and GOCI (divided by 8). Note that to eliminate the cloud cover differences in the spatial and temporal domains, the statistics of each factor was restricted to small windows with a size of 10 × 10 pixels (see locations in Figure 6). The last three rows list the results for the tongue-shaped low DPVOs zones in winter.

		Atmospheric Correction Failure	High Sunglint	Cloud Cover	High Solar Zenith Angle	Chlorophyll Algorithm Failure	High Sensor Zenith Angle	Straylight	High Radiance	DPVOs	Total Invalid Data
Inshore	MODISA	0.04%	0.04%	54.92%	0.00%	0.00%	18.37%	7.15%	18.50%	0.65%	99.04%
	GOCI	0.01%	0.01%	92.11%	4.90%	0.08%	0.00%	0.00%	0.00%	6.92%	97.10%
	GOCI_MODISA_	0.01%	0.01%	92.19%	0.00%	0.06%	0.00%	0.00%	0.00%	7.83%	92.26%
Offshore	MODISA	0.00%	0.00%	50.06%	0.00%	0.01%	16.72%	16.90%	14.67%	3.31%	98.37%
	GOCI	0.02%	0.02%	81.30%	4.85%	0.79%	0.00%	0.00%	0.00%	17.11%	86.97%
	GOCI_MODISA_	0.01%	0.01%	81.01%	0.17%	0.80%	0.00%	0.00%	0.00%	18.40%	82.01%
Within the tongue-shaped zone	MODISA	0.00%	0.00%	53.84%	0.00%	0.00%	17.06%	12.58%	15.73%	0.37%	99.22%
	GOCI	0.01%	0.01%	93.25%	4.66%	0.12%	0.00%	0.00%	0.00%	5.40%	98.04%
	GOCI_MODISA_	0.00%	0.00%	92.50%	0.43%	0.12%	0.00%	0.00%	0.00%	6.29%	93.06%

## References

[1] McClain C.R. (2009). A decade of satellite ocean color observations. Annu. Rev. Mar. Sci..

[2] Yoder J., Kennelly M. (2006). What Have We Learned About Ocean Variability from Satellite Ocean Color Imagers?. Oceanography.

[3] Feng L., Hu C. (2015). Comparison of Valid Ocean Observations Between MODIS Terra and Aqua Over the Global Oceans. IEEE Trans. Geosci. Remote Sens..

[4] Arnone R., Vandermuelen R., Soto I., Ladner S., Ondrusek M., Yang H. (2017). Diurnal changes in ocean color sensed in satellite imagery. J. Appl. Remote Sens..

[5] Lou X., Hu C. (2014). Diurnal changes of a harmful algal bloom in the East China Sea: Observations from GOCI. Remote Sens. Environ..

[6] Son Y.B., Choi B.-J., Kim Y.H., Park Y.-G. (2015). Tracing floating green algae blooms in the Yellow Sea and the East China Sea using GOCI satellite data and Lagrangian transport simulations. Remote Sens. Environ..

[7] Qi L., Hu C., Visser P.M., Ma R. (2018). Diurnal changes of cyanobacteria blooms in Taihu Lake as derived from GOCI observations. Limnol. Oceanogr..

[8] Jiang L., Wang M. (2016). Diurnal Currents in the Bohai Sea Derived from the Korean Geostationary Ocean Color Imager. IEEE Trans. Geosci. Remote Sens..

[9] Park K.-A., Lee M.-S., Park J.-E., Ullman D., Cornillon P.C., Park Y.-J. (2018). Surface currents from hourly variations of suspended particulate matter from Geostationary Ocean Color Imager data. Int. J. Remote Sens..

[10] Pan Y., Shen F., Wei X. (2018). Fusion of Landsat-8/OLI and GOCI Data for Hourly Mapping of Suspended Particulate Matter at High Spatial Resolution: A Case Study in the Yangtze (Changjiang) Estuary. Remote Sens..

[11] Yan Y., Huang K., Shao D., Xu Y., Gu W. (2019). Monitoring the Characteristics of the Bohai Sea Ice Using High-Resolution Geostationary Ocean Color Imager (GOCI) Data. Sustainability.

[12] Yan Y., Uotila P., Huang K., Gu W. (2020). Variability of sea ice area in the Bohai Sea from 1958 to 2015. Sci. Total. Environ..

[13] Murakami H. (2016). Ocean Color Estimation by Himawari-8/Ahi. SPIE Asia-Pac. Remote Sens. SPIE.

[14] Chen X., Shang S., Lee Z., Qi L., Yan J., Li Y. (2019). High-frequency observation of floating algae from AHI on Himawari-8. Remote Sens. Environ..

[15] Hu C., Feng L. (2014). GOES Imager Shows Diurnal Changes of a Trichodesmium erythraeum Bloom on the West Florida Shelf. IEEE Geosci. Remote Sens. Lett..

[16] Yang C.-S., Song J.-H. (2012). Geometric performance evaluation of the Geostationary Ocean Color Imager. Ocean Sci. J..

[17] Ryu J.-H., Han H.-J., Cho S., Park Y.-J., Ahn Y.-H. (2012). Overview of geostationary ocean color imager (GOCI) and GOCI data processing system (GDPS). Ocean Sci. J..

[18] Fishman J., Iraci L.T., Al-Saadi J., Chance K., Chavez F., Chin M., Coble P., Davis C., Digiacomo P.M., Edwards D. (2012). The United States’ Next Generation of Atmospheric Composition and Coastal Ecosystem Measurements: NASA’s Geostationary Coastal and Air Pollution Events (GEO-CAPE) Mission. Bull. Am. Meteorol. Soc..

[19] Feng L., Hu C., Barnes B.B., Mannino A., Heidinger A., Strabala K., Iraci L.T. (2017). Cloud and Sun-glint statistics derived from GOES and MODIS observations over the Intra-Americas Sea for GEO-CAPE mission planning. J. Geophys. Res. Atmos..

[20] He X., Stamnes K., Bai Y., Li W., Wang D. (2018). Effects of Earth curvature on atmospheric correction for ocean color remote sensing. Remote Sens. Environ..

[21] King M.D., Ackerman S., Hubanks P.A., Platnick S., Menzel W.P. (2013). Spatial and Temporal Distribution of Clouds Observed by MODIS Onboard the Terra and Aqua Satellites. IEEE Trans. Geosci. Remote Sens..

[22] He X., Bai Y., Pan D., Huang N., Dong X., Chen J., Chen C.-T.A., Cui Q. (2013). Using geostationary satellite ocean color data to map the diurnal dynamics of suspended particulate matter in coastal waters. Remote Sens. Environ..

[23] Choi J.-K., Min J.-E., Noh J.H., Han T.-H., Yoon S., Park Y.J., Moon J.-E., Ahn J.-H., Ahn S.-M., Park J.-H. (2014). Harmful algal bloom (HAB) in the East Sea identified by the Geostationary Ocean Color Imager (GOCI). Harmful Algae.

[24] Kim H., Son Y.B., Jo Y.-H. (2018). Hourly Observed Internal Waves by Geostationary Ocean Color Imagery in the East/Japan Sea. J. Atmospheric Ocean. Technol..

[25] Concha J., Mannino A., Franz B.A., Kim W. (2019). Uncertainties in the Geostationary Ocean Color Imager (GOCI) Remote Sensing Reflectance for Assessing Diurnal Variability of Biogeochemical Processes. Remote Sens..

[26] Neveux J., Dupouy C., Blanchot J., Le Bouteiller A., Landry M.R., Brown S.L. (2003). Diel Dynamics of Chlorophylls in High-Nutrient, Low-Chlorophyll Waters of the Equatorial Pacific (180°): Interactions of Growth, Grazing, Physiological Responses, and Mixing. J. Geophys. Res. Oceans.

[27] MacIntyre H., Kana T.M., Anning T., Geider R.J. (2002). Photoacclimation of Photosynthesis Irradiance Response Curves and Photosynthetic Pigments in Microalgae and Cyanobacteria. J. Phycol..

[28] Mercado J.M., Ramírez T., Cortés D., Sebastián M., Reul A., Bautista B. (2006). Diurnal changes in the bio-optical properties of the phytoplankton in the Alborán Sea (Mediterranean Sea). Estuarine Coast. Shelf Sci..

[29] Tang J., Wang X., Song Q., Li T., Chen J., Huang H., Ren J. (2004). The Statistic Inversion Algorithms of Water Constituents for the Huanghai Sea and the East China Sea. Acta Oceanologica Sinica.

[30] Otten T.G., Xu H., Qin B., Zhu G., Paerl H.W. (2012). Spatiotemporal Patterns and Ecophysiology of ToxigenicMicrocystisBlooms in Lake Taihu, China: Implications for Water Quality Management. Environ. Sci. Technol..

[31] Carlson R.E. (1977). A trophic state index for lakes. Limnol. Oceanogr..

[32] Kim W., Moon J.-E., Park Y.-J., Ishizaka J. (2016). Evaluation of chlorophyll retrievals from Geostationary Ocean Color Imager (GOCI) for the North-East Asian region. Remote Sens. Environ..

[33] Hu C., Lee Z., Franz B. (2012). Chlorophyll Aalgorithms for Oligotrophic Oceans: A Novel Approach Based on Three-Band Reflectance Difference. J. Geophys. Res. Oceans.

[34] O’Reilly J.E., Maritorena S., Mitchell B.G., Carder K.L., Garver S.A., Kahru M., McClain C., Siegel D.A. (1998). Ocean color chlorophyll algorithms for SeaWiFS. J. Geophys. Res. Space Phys..

[35] Hooker B.S., Firestone E.R., Esaias W.E., Feldman G.C., Gregg W.W., Mcclain C.R. (1992). An Overview of Seawifs and Ocean Color.

[36] Feng L., Hu C. (2016). Cloud adjacency effects on top-of-atmosphere radiance and ocean color data products: A statistical assessment. Remote Sens. Environ..

[37] Feng L., Hu C. (2017). Land adjacency effects on MODIS Aqua top-of-atmosphere radiance in the shortwave infrared: Statistical assessment and correction. J. Geophys. Res. Oceans.

[38] Hu C., Feng L., Lee Z., Franz B., Bailey S.W., Werdell P.J., Proctor C.W., Werdell J. (2019). Improving Satellite Global Chlorophyll a Data Products Through Algorithm Refinement and Data Recovery. J. Geophys. Res. Oceans.

[39] Le Borgne R., Rodier M. (1997). Net zooplankton and the biological pump: A comparison between the oligotrophic and mesotrophic equatorial Pacific. Deep. Sea Res. Part II Top. Stud. Oceanogr..

[40] Lohrenz S., Weidemann A.D., Tuel M. (2003). Phytoplankton spectral absorption as influenced by community size structure and pigment composition. J. Plankton Res..

[41] Babin M., Stramski D., Ferrari G.M., Claustre H., Bricaud A., Obolensky G., Hoepffner N. (2003). Variations in the light absorption coefficients of phytoplankton, nonalgal particles, and dissolved organic matter in coastal waters around Europe. J. Geophys. Res. Space Phys..

[42] Fujiki T., Taguchi S. (2002). Variability in chlorophyll a specific absorption coefficient in marine phytoplankton as a function of cell size and irradiance. J. Plankton Res..

[43] Ding K., Gordon H.R. (1994). Atmospheric correction of ocean-color sensors: Effects of the Earth’s curvature. Appl. Opt..

[44] Kim W., Ahn J.-H., Park Y.-J. (2015). Correction of Stray-Light-Driven Interslot Radiometric Discrepancy (ISRD) Present in Radiometric Products of Geostationary Ocean Color Imager (GOCI). IEEE Trans. Geosci. Remote Sens..

[45] Hu C., Feng L., Lee Z., Davis C.O., Mannino A., McClain C.R., Franz B.A. (2012). Dynamic range and sensitivity requirements of satellite ocean color sensors: Learning from the past. Appl. Opt..

[46] Lin G., Yang Q., Wang Y., Lin W. (2012). Species Composition and Distribution Characteristics of Phytoplankton in Northern Sea of Fujian, China During Withdraw of Zhe-Min Coastal Current. Chin. J. Appl. Environ. Biol..

[47] McClain C.R., Signorini S.R., Christian J.R. (2004). Subtropical gyre variability observed by ocean-color satellites. Deep. Sea Res. Part II Top. Stud. Oceanogr..

[48] Rykaczewski R.R., Dunne J.P. (2011). A measured look at ocean chlorophyll trends. Nature.

[49] Zhang C., Shang S., Chen D., Shang S. (2005). Short-Term Variability of the Distribution of Zhe-Min Coastal Water and Wind Forcing During Winter Monsoon in the Taiwan Strait. J. Remote Sens..

[50] Gordon H.R. (1997). Atmospheric correction of ocean color imagery in the Earth Observing System era. J. Geophys. Res. Space Phys..

[51] Gordon H.R., Wang M. (1994). Retrieval of water-leaving radiance and aerosol optical thickness over the oceans with SeaWiFS: A preliminary algorithm. Appl. Opt..

[52] Wang M., Ahn J.-H., Jiang L., Shi W., Son S., Park Y.-J., Ryu J.-H. (2013). Ocean color products from the Korean Geostationary Ocean Color Imager (GOCI). Opt. Express.

[53] Maritorena S., Siegel D.A., Peterson A.R. (2002). Optimization of a semianalytical ocean color model for global-scale applications. Appl. Opt..

[54] Bailey S.W., Werdell P.J. (2006). A multi-sensor approach for the on-orbit validation of ocean color satellite data products. Remote Sens. Environ..

